# A Patient with Posterior Cortical Atrophy Possesses a Novel Mutation in the Presenilin 1 Gene

**DOI:** 10.1371/journal.pone.0061074

**Published:** 2013-04-12

**Authors:** Emilia J. Sitek, Ewa Narożańska, Beata Pepłońska, Sławomir Filipek, Anna Barczak, Maria Styczyńska, Krzysztof Mlynarczyk, Bogna Brockhuis, Erik Portelius, Dorota Religa, Maria Barcikowska, Jarosław Sławek, Cezary Żekanowski

**Affiliations:** 1 Department of Neurological and Psychiatric Nursing, Medical University of Gdansk, Gdansk, Poland; 2 Department of Neurology, St. Adalbert Hospital, Gdansk, Poland; 3 Department of Neurodegenerative Disorders, Mossakowski Medical Research Centre, Polish Academy of Sciences, Warszawa, Poland; 4 Faculty of Chemistry, University of Warsaw, Warszawa, Poland; 5 Department of Neurodegenerative Diseases, Neurology Clinic, MSWiA Hospital, Warszawa, Poland; 6 Department of Nuclear Medicine, Medical University, Gdansk, Poland; 7 Clinical Neurochemistry Laboratory, Institute of Neuroscience and Physiology, Department of Psychiatry and Neurochemistry, The Sahlgrenska Academy, University of Gothenburg, Gothenburg, Sweden; 8 Department of Neurobiology, Care Sciences and Society, Karolinska Institutet, Huddinge, Sweden; Louisiana State University Health Sciences Center, United States of America

## Abstract

Posterior cortical atrophy is a dementia syndrome with symptoms of cortical visual dysfunction, associated with amyloid plaques and neurofibrillary tangles predominantly affecting visual association cortex. Most patients diagnosed with posterior cortical atrophy will finally develop a typical Alzheimer's disease. However, there are a variety of neuropathological processes, which could lead towards a clinical presentation of posterior cortical atrophy. Mutations in the presenilin 1 gene, affecting the function of γ-secretase, are the most common genetic cause of familial, early-onset Alzheimer's disease. Here we present a patient with a clinical diagnosis of posterior cortical atrophy who harbors a novel Presenilin 1 mutation (I211M). *In silico* analysis predicts that the mutation could influence the interaction between presenilin 1 and presenilin1 enhancer-2 protein, a protein partner within the γ-secretase complex. These findings along with published literature support the inclusion of posterior cortical atrophy on the Alzheimer's disease spectrum.

## Introduction

Posterior cortical atrophy (PCA) is a progressive neurodegenerative disorder of higher visual processing, both in terms of object and space perception [1], [2]. At autopsy, a majority of the PCA patients are diagnosed with Alzheimer's disease (AD) due to similar pathology including neuronal loss and accumulation of amyloid plaques, but with the special distribution to visual cortex [3]. The most PCA cases have intact episodic memory and insight early in the course of the disease, which enables differential diagnosis with typical (amnestic) AD [4], [5].

For many years PCA has been regarded as an atypical visual variant of AD [6] or a distinct nosological entity such as non-AD pathologies including Lewy body disease, corticobasal degeneration and prion disease [7], [8], [9], [10]. Recent epidemiological studies indicate that less than 5% of patients with AD are also affected with PCA [11].

PCA usually display the same cerebrospinal fluid (CSF) biomarker signature as AD, including elevated levels of total tau (t-tau) and phosphorylated tau (p-tau) and decreased levels of amyloid-β (Aβ) consisting of 42 amino acids (Aβ_42_). However, some studies have shown that a significant proportion of PCA and primary progressive non-fluent or logopenic aphasia patients may have atypical t-tau/Aβ_42_ and p-tau/Aβ_42_ profiles [12], [13], [14].

The genetic basis of PCA remains elusive [1], [15], usually not showing autosomal dominant inheritance patterns and generally there is no family history of dementia [16]. This is in contrast to early-onset autosomal dominant AD, where mutations in the presenilin 1 gene (*PSEN1*) are the most common cause of familial AD (FAD). Patients bearing specific mutations may present different clinical phenotypes. Some patients with mutations in pesenilin 1 or presenilin 2 (*PSEN2*) genes are clinically diagnosed with frontotemporal dementia or other neurodegenerative conditions [17], [18]. Recently Crutch and colleagues reported on a PCA phenotype, which is associated with autosomal dominant FAD [1]. More recently it was shown that a mutation in prion protein gene (*PRNP*), or *PSEN1* gene (Q223R) were associated with PCA phenotype [19], [20].

Here we present a patient with clinically diagnosed PCA harboring a I211M mutation in *PSEN1*. *In silico* analysis, indicates that the I211M mutation could change the interaction between presenilin 1 (PS1) and the PS1 enhancer-2 protein (PEN-2), its partner within the γ-secretase complex.

## Materials and Methods

### Case report

A 67 year-old right-handed woman with university education reported a three-year history of progressive visual problems. Medical history included myopia, arterial hypertension, radioiodine treatment for nontoxic multinodular goiter and diabetes type 2 (diagnosed at the age of 64). Her family history suggested several problems including managing objects in the near, as well as distant space. Because of visual problems, she turned to listening to the radio instead of watching TV. In the kitchen she recognized objects with touch due to the partially preserved spatial memory. She also experienced severe difficulties when eating and mild difficulties in dressing. She was unable to recognize faces, but compensated for this difficulty by correct voice recognition. In new surroundings, she had difficulties to orientate. Before hospitalization at the Neurology Department she continued to live on her own and was independent in the daily activities, except those in which accurate vision was indispensable and compensatory strategies were ineffective.

**Figure 1:**
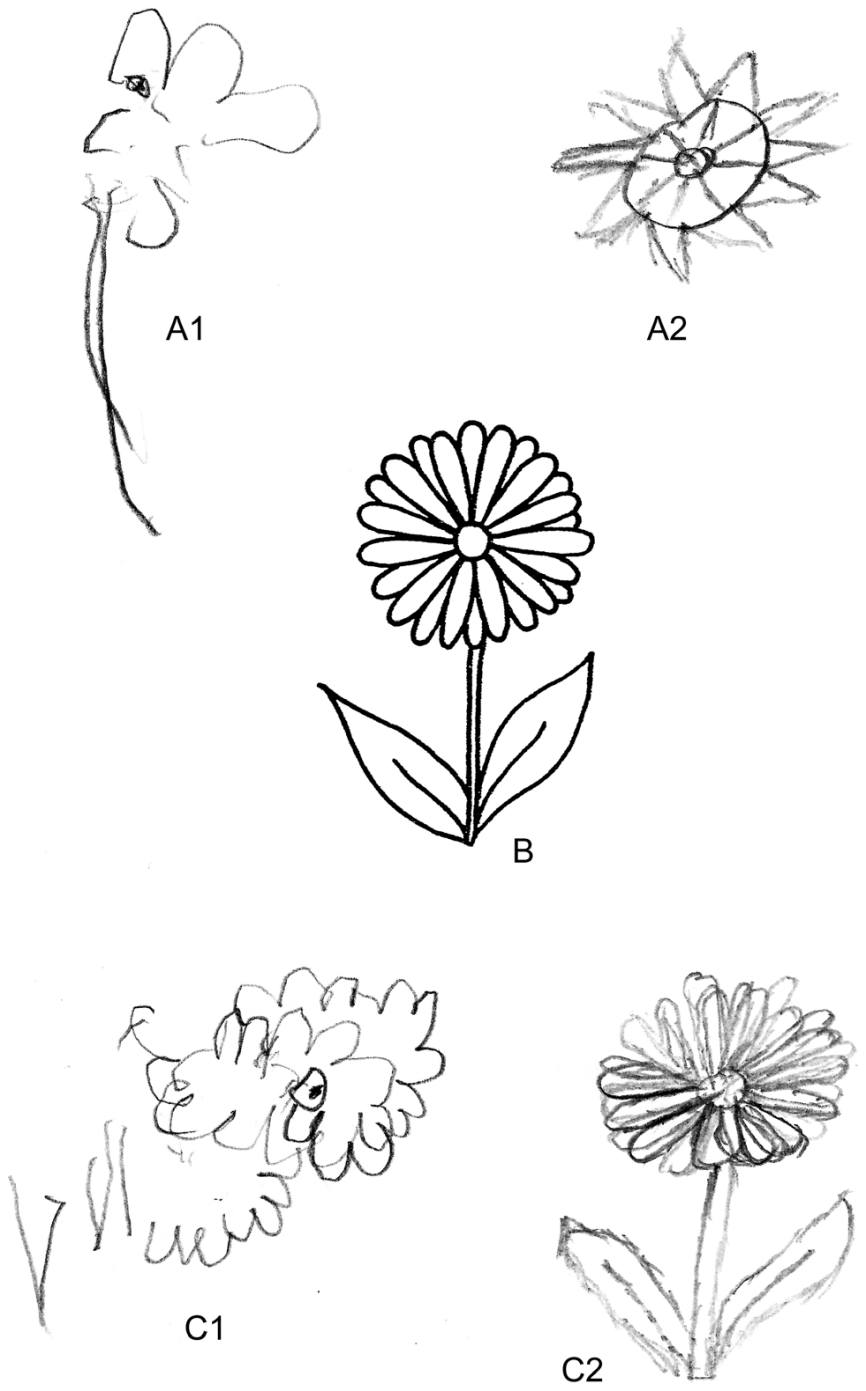
Analysis of visuospatial dysfunction of the patient. Patient's drawings in the context of drawings by a patient with semantic dementia. (SD) scoring 19 in MMSE were presented. A) flowers drawn by the patients from memory: A1) PCA patient, A2) SD patient; B) model, C) patients' copy: C1) PCA patient, C2) SD patient. Copying pictures and drawing to command indicated severe optic ataxia and partial simultanognosia. The test was performed at first neuropsychological assessment. During the second assessment (six months later) the patient was unable to draw even simple patterns and presented with complete simultanognosia.

Neurological and neuropsychological examination revealed no abnormalities apart from visual agnosia, optic ataxia, simultagnosia, prosopagnosia, unilateral neglect and alexia. A screening neuropsychological examination, as well as an interview with the proband's family (respectively: Mini Mental State Examination, MMSE 21/30, and Blessed Dementia Rating Scale, BDRS 4.5) indicated mild dementia. Neuropsychological assessment confirmed both the core and supporting symptoms of PCA. Visual deficits involved both object and space perception. The clock face filling test revealed severe optic ataxia, the Rey Complex Figure Test score was <10 percentile. The visuospatial dysfunction predominated in the clinical picture (Figure 1), while episodic memory, executive and language functions were mostly preserved. A comparison of copy/drawing to command provided clinically important information on the relatively well-preserved semantic knowledge (in comparison to semantic dementia), degree of optic ataxia, simultanagnosia and unilateral neglect. The patient had a preserved insight into her visual deficits which resulted in searching for compensatory strategies, and exhibited anxiety associated with the progressive nature of the disorder, albeit no depression (Montgomery Asberg Depression Rating Scale, MADRS 5).

A second neuropsychological evaluation six months later showed worsening of the visuospatial function and praxis and mild deficits of verbal memory and speech comprehension. The profile was still consistent with PCA and showed the predominant progression of visual deficits also in line with PCA diagnosis.

The third neuropsychological evaluation 2 years after the first assessment showed the global deterioration of cognitive status (MMSE 15, BDRS 9.5), and increasing of the visual deficits, both of visual agnosia and visuospatial dysfunction. The marked deterioration of visual function was accompanied by less severe, albeit significant, episodic memory decline and language impairment suggestive of transcortical sensory aphasia. Executive function was only mildly impaired. The patient's anxiety and depressive symptoms increased over time (MADRS 16). The patient's disability was still mostly due to visual dysfunction.

**Figure 2:**
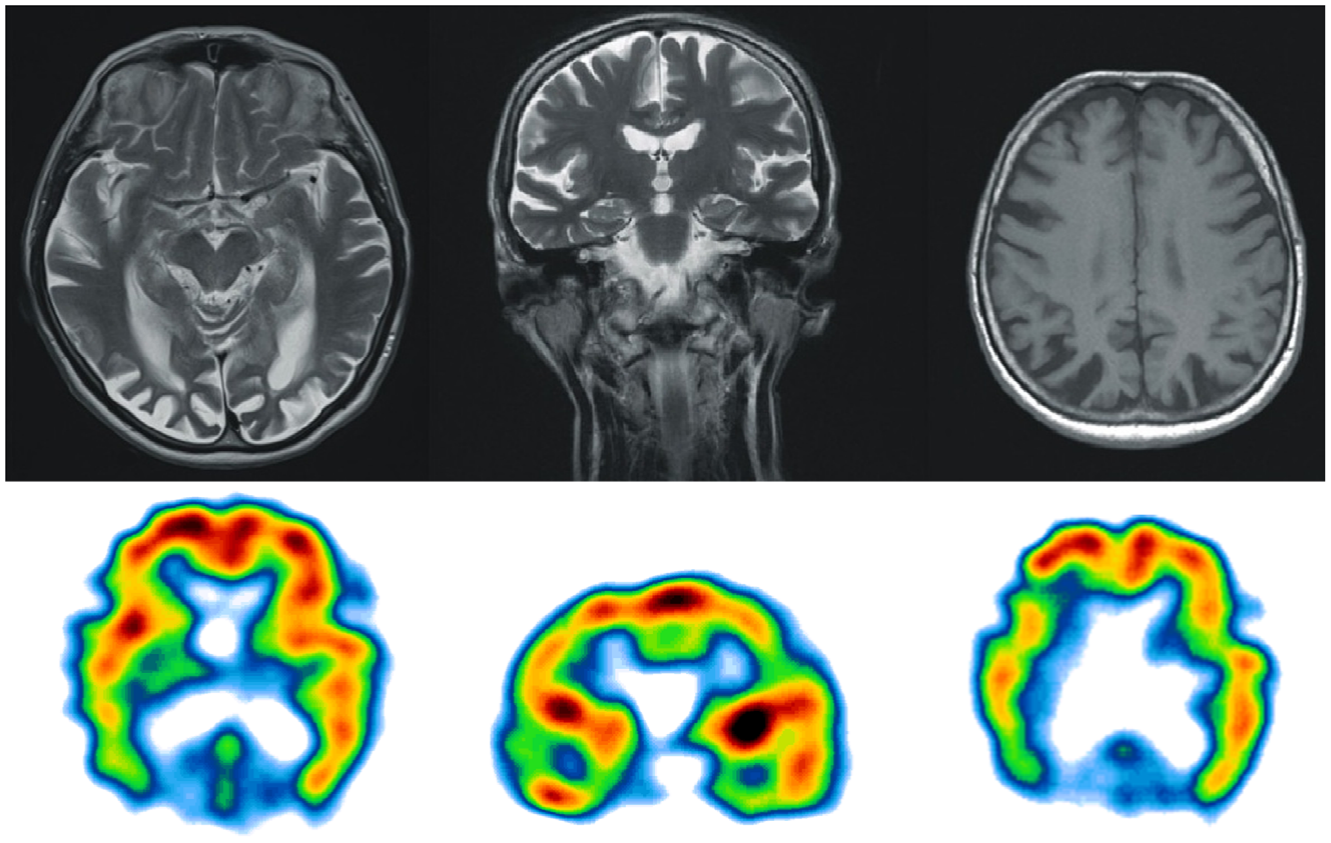
Results of MRI and SPECT examinations of the proband. Magnetic Resonance Imaging revealed marked cortical and subcortical atrophy within both occipital and parietal lobes bilaterally. The atrophy was less pronounced in the frontal and temporal lobes, and the hippocampal structures of the temporal lobes were mostly preserved. Single Photon Emission Computed Tomography demonstrated severe hypoperfusion within the parietal, occipital and temporal lobes bilaterally.

**Figure 3:**
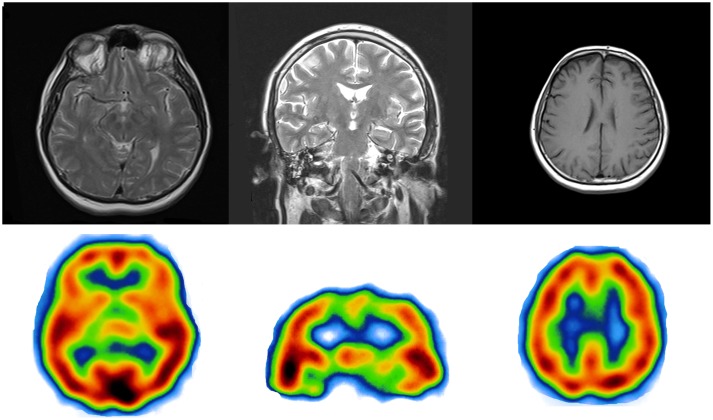
Results of MRI and SPECT examination of a control subject. Magnetic Resonance Imaging and Single Photon Emission Computed Tomography demonstrating the normal scans of a control, age-matched patient with no signs of any neurodegenerative disorder.

Magnetic resonance imaging (MRI) at the age of 67 revealed marked cortical and subcortical atrophy within both occipital and parietal lobes bilaterally. The atrophy was greater in the former. The right parietal lobe was more atrophic than the left one, with no asymmetry in the occipital lobes. The atrophy was less pronounced in the frontal and temporal lobes, and the hippocampal structures of the temporal lobes were mostly preserved. Single Photon Emission Computed Tomography (SPECT) demonstrated severe hypoperfusion within the parietal, occipital and temporal lobes bilaterally (Figure 2; compare with Figure 3 showing MRI/SPECT brain images from a control subject). Visual evoked potentials test showed reduced amplitude of P100 with prolonged latency (138 ms) on the left and normal amplitude with prolonged latency on the right (123 ms). The confrontation visual field examination revealed severe peripheral deficits bilaterally with central field vision preservation. Visual acuity tests using a Snellen chart showed myopia (Vod and Vos = 0.3). Intraocular pressure and dilated fundus examination were normal.

The family history of the patient revealed no records of dementia, however, her father died at the age of 51, due to stroke, however no post-mortem examination was performed. The proband's mother died at 80 due to leukemia, with no neurological problems recorded. An older brother of the patient died at the age of 78 due to a prostate cancer. An 82-year-old sister of the proband is neurologically healthy, and is not a carrier of the I211M mutation. Two children of the proband, aged 33 and 37 are healthy.

A written, signed consent was obtained from the proband and the family members. The genetic study was approved by the Ethics Committee of the CSK-MSWiA Hospital (Warszawa, Poland) in compliance with the national legislation and the Code of Ethical Principles for Medical Research Involving Human Subjects of the World Medical Association.

### Methods

DNA was isolated from peripheral blood leucocytes of the proband and the family members using standard procedures. Intronic primers were used to amplify and sequence exons 3–12 of PSEN1, exons 16 and 17 of APP, exons 1–12 of PSEN2, and PRNP generally as described previously [17], [21]. The absence of the mutation was confirmed in a group of 210 healthy subjects aged >65 (using RFLP method), and 170 early-onset AD patients (using fluorescent sequencing) from the Polish population. A written informed consent was obtained from all individuals.

The putative impact of the mutation on the transmembrane domains of PS1 was assessed using bioinformatic and *in silico* modeling approaches.

To elucidate an interface between human PS1 and PEN-2, residues 186–250 of PS1 (TM4–TM5) and 15–41 (TM1) of PEN-2 which are assumed to contain the transmembrane regions were investigated. The transmembrane part of PEN-2 was constructed as α-helix while the PS1 TM4–TM5 segment, including a loop, was modeled using membrane *ab initio* application from Rosetta package (v3.4) [22]. The resulting helices were antiparallel and not crossed. Since the experimental data show that the WNF motif in PS1 TM4 is crucial for binding [23], [24] we used this side of TM4 as a part of a binding site with Pen-2. Residue N33, which was shown to be a part of ER retention signal, is also involved in the binding process on PEN-2 side [25], [26]. The initial structures of the complex were pre-processed using a docking prepack protocol and subjected to a local docking procedure [27]. The best scored 200 of 50000 structures were clustered and filtered for correct orientation of both PS1 and PS2 fragments and for mutual positions of N204 and N33 residues. Selected structures of the complex characterized with the highest number of interactions in the interface were chosen for short 5 ns molecular dynamics refinement in POPC (1-palmitoyl-2-oleoyl-sn-glycero-3-phosphocholine) membrane. All simulations were conducted in Yasara program employing Amber03 force field [28], [29].

The CSF levels of amyloid peptides (Aβ42 and Aβ40) were determined using the Aβ Triplex assay (Human Aβ peptide Ultra-Sensitive Kits) provided and developed by Meso Scale Discovery (MSD, Gaithersburg, Maryland, USA) as described elsewhere [30]. Briefly, this assay uses C-terminus-specific antibodies to capture the different Aβ peptides and a SULFO-TAG-labeled anti-Aβ antibody (4G8) for detection with electrochemiluminescence. The CSF levels of total tau (t-tau), and tau phosphorylated at threonine 181 (p-tau) were determined using xMAP technology, as previously described [31].

For MesoScale (Karolinska Institute & University of Gothenburg, Sweden) measurements the cut-off ranges for healthy controls were Aβ_42_ 400–1200 pg/ml and Aβ_40_ 7000–15000 pg/ml). For xMAP measurements the cut-off values for p-tau was >70 pg/ml, and for t-tau >400 pg/ml.

## Results

A patient with clinically diagnosed PCA, harboring a novel ATT>ATG mutation at codon 211 (I211M, g.44652T>G) of *PSEN1* was identified. No other mutations were found in the *PSEN1*, *PSEN2*, *APP* or *PRNP* genes, except for already described polymorphisms. The proband is a carrier of the E3/E3 *APOE* genotype. The I211M mutation was absent in a series of 210 control subjects and 170 early-onset AD cases.

**Figure 4:**
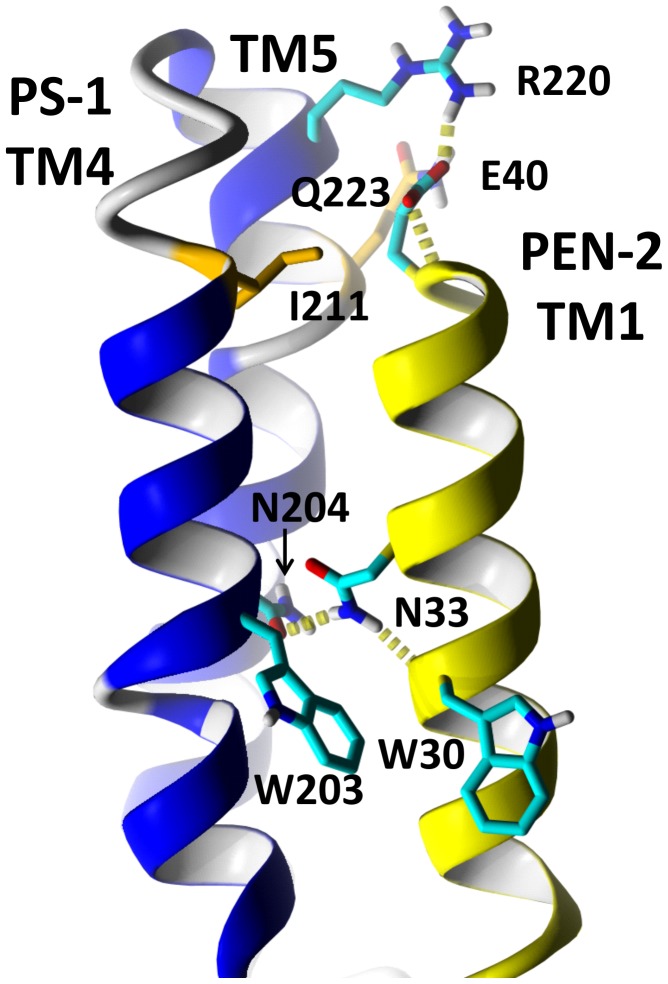
In silico modeling of presenilin 1 and PEN-2 protein interaction. A model of a complex of PS1 TM4-TM5 (in blue) and PEN-2 TM1 (in yellow) were shown. Only the residues discussed in the text are depicted and colored: I211 and Q223 in orange other residues in cyan. Hydrogen bonds are visualized as dashed yellow cylinders. The residues N204 and N33 form a hydrogen bond, while R220 and E40 form a salt bridge at extracellular side of the presenilin 1.

We performed an *in silico* analysis to investigate the impact of the I211M substitution on the PS1 architecture and its interactions with other protein partners within γ-secretase complex. The mutation is located in the last turn of the cytoplasmic part of transmembrane helix 4 (TM4). The residue faces the membrane part but also TM3 of PS1 according to a previously presented model [24]. Only residues facing the interior of a protein are assumed to have no direct effect on the protein's interactions with other entities. Employing the docking technique followed by molecular dynamics in explicit POPC membrane we obtained a model of a protein complex between PS1 TM4–TM5 and PEN-2 TM1. The important residues found to form an interface with PEN-2, W203 and N204, were found to be in contact with PEN-2 TM1 (Figure 4) and there was a hydrogen bond between N204 and N33 which was stable during the simulation. At the extracellular side the residues R220 and E40 formed a salt bridge. The AD residues I211 and Q223 are located nearly in the same environment in contact with this salt bridge R220-E40.

The proband presents Aβ signature atypical for AD: Aβ_42_ (787 pg/ml) and Aβ_40_ (14599 pg/ml) levels are in the range of healthy controls. In contrast, the elevated p-tau (97 pg/ml), and slightly elevated t-tau (418 pg/ml) levels are consistent with an AD signature.

## Discussion

PCA is a descriptive term due to a lack of consistency between studies regarding the classification of PCA at the disease and syndrome level [1]. Recent recommendations from the National Institute of Aging and the Alzheimer's Association (NIA–AA) workgroup include different non-amnestic symptoms such as visuospatial presentation as a possible core clinical criterion of atypical AD [10]. Atypical presentations and specific patterns of atrophy are frequently associated with early onset. However, little is known about risk factors. Genetic and/or environmental background seems to predispose patients to early-onset AD (EOAD) [32]. Some authors have suggested that it could be due to different age-related vulnerability to amyloid toxicity [33], [34].

In the PCA patient presented here the structural and functional neuroimaging data correlated with the observed clinical features, reflecting dysfunction of both dorsal and ventral visual streams, which is quite common in PCA [35]. However, the predominance of ventral stream dysfunction was evidenced by prominent visual object agnosia, alexia and less pronounced spatial disorientation. It corresponded with more severe atrophy and hypoperfusion in the occipital lobes than in the parietal lobes. Moreover, despite the global deficits in spatial attention, features of left-sided unilateral neglect were noticed in visual search trials and even in drawings from memory. Such hemispatial neglect was in accordance with the more pronounced right-sided parietal atrophy and hypoperfusion.

The I211M mutation could be causative, as the mutation was absent in large groups of control subjects and early-onset AD cases from the Polish population. The I211M mutation was also absent in public DNA sequence variation databases, including Archive EnsEMBL (release 69, October 2012). The mutation was absent in the 82-year-old proband's sister, presenting no neurological symptoms. It could be speculated that the mutation – if fully penetrant - was inherited from the proband's father (who died before the putative age of onset connected with the mutation), and not from the mother (who reached 80 years of age with no neurological symptoms).

Also an *in silico* analysis of the I211M mutation suggests that the mutation is functional. Firstly, I211M changes the hydrophobic isoleucine into the nonpolar methionine and thus potentially could be tolerated without changing the global transmembrane domain structure. Analysis of the protein variant sequence using Sorting Intolerant From Tolerant (SIFT) program (http://sift.jcvi.org/), which predicts whether an amino acid substitution affects the protein function based on sequence homology and the physical properties of amino acids, suggests that methionine could be tolerated in position 211. However, analyses using ConSeq and ConSurf programs (http://consurftest.tau.ac.il/) suggest that I211 residue is highly conserved in evolution and functional.

To make our claim on the true disease-causing nature of the mutation more likely we performed an *in silico* analysis of the impact of the I211M substitution on PS1 architecture. We also included residue Q223, which substitution (Q223R) is associated with visual agnosia and cortical atrophy phenotype [20]. The obtained results strongly suggest that the mutated residue 211, as well residue 223, could affect the interaction with its protein partners within the γ-secretase complex. PS-1 only functions within the γ-secretase complex through its interactions with PEN-2 and two other membrane components: nicastrin and anterior pharynx defective-1 (APH-1) [36]. It was previously proposed that the TM4 helix, encompassing I211 residue, is the binding site for PEN-2 [37]. The NF motif within this domain, which is critical for PEN-2 binding, precedes the I211 position only by two helix turns. The important residues W203 and N204, found to form an interface with PEN-2, were also found to be in contact with PEN-2 TM1 and there was a hydrogen bond between N204 and N33 which was stable during the simulation. At the extracellular side the residues R220 and E40 formed a salt bridge. The residues I211 and Q223, mutated in patients with PCA or PCA-like phenotypes, are located nearly in the same environment and are in contact with the R220-E40 salt bridge. Therefore, mutations of these residues most probably could influence binding of the same region of PEN-2 and possibly can result in a similar clinical phenotype of AD, as it was recently shown in case of Q223R [20]. However, it should be noted that the Q223R mutation was identified for the first time in an EOAD patient with spastic paraplegia [38].

It was recently proposed that over-expression of PEN-2 or PS1 mutant *in vitro* altered the equilibrium between PS1- and PS2-γ-secretases, resulting in a profound effect on the enzyme activity and the Aβ_42_∶Aβ_40_ ratio [39]. It could be speculated that I211M-mutated PS1, due to the conformational changes in PS1, has a reduced ability to compete with native PS1 and PS2 for PEN-2, therefore favoring the PS2 γ-secretase complex formation. This could result in a reduction of the overall γ-secretase activity and an increase of the Aβ_42_∶Aβ_40_ ratio [36]. It could be further speculated that the neurons, selectively damaged in PCA, could be those particularly vulnerable to the disruption of the equilibrium between PS1- and PS2-containing γ-secretases [40].

To test this hypothesis we quantified the core CSF biomarkers for AD. The proband presents a biomarker signature atypical for AD, albeit, with elevated p-tau (97 pg/ml) and slightly elevated t-tau (418 pg/ml) levels, which is consistent with an AD signature. For instance the Q223R mutation identified in a patient diagnosed with AD with spastic paraparesis displayed a biomarker profile which was different from the profile of our patient i.e. low Aβ_42_ and normal t-tau [38]. Furthermore, a patient with the same mutation and PCA-like phenotype had decreased Aβ_42_ levels together with increased t-tau and p-tau [20]. Also in a recently published study of 22 patients with clinical diagnosis of PCA, in four cases Aβ_42_ and t-tau, p-tau levels were not typical for AD [41]. Therefore, the atypical CSF biomarkers presentation should not exclude the possibility of causative link between PCA and AD.

Moreover, the profile of cognitive deterioration of the proband supports the assumption that PCA phenotype should be interpreted within AD spectrum. Despite the predominance of visual dysfunction throughout the 2-year observation period, in the follow-up testing episodic memory decline, was evident.

The putative influence of I211M mutation on PS1:PEN-2 interaction and on γ-secretase activity as a result, is supported by the recent findings that Pen-2 could directly modulate the pore-like structure around the catalytic center formed by PS1 transmembrane domains [42].

In summary, we report a novel PSEN1 I211M mutation, which could be causally connected with the PCA phenotype. It could further be speculated that the clinical phenotype could be a result of aberrant APP processing, as well as other altered functions of PS1.
